# A 2025 Literature Review on Arrhythmias: Returning the Focus to Basic Science

**DOI:** 10.19102/icrm.2025.17016

**Published:** 2026-01-15

**Authors:** Sara Bushmen, Emily Collins, Andy C. Kiser

**Affiliations:** 1 School of Cardiovascular Perfusion, Carlow University, Pittsburgh, PA, USA; 2 Cardiovascular Services, St. Clair Health, Pittsburgh, PA, USA; 3 Department of Cardiothoracic Surgery, University of Pittsburgh School of Medicine, Pittsburgh, PA, USA

It is a well-established norm in the research community to find inspiration in technological advances, novel techniques, pharmacological developments, and innovative procedures. This past year, however, offered an element of basic science research. Reports in 2025 included publications investigating arrhythmias at the cellular level. Examination of mitochondrial dysfunction, immune system reactions, and gene modulation reminds us that research in these areas, focusing on the cellular origins of cardiac arrhythmias, remains consequential for future treatment options.

## Immune cells and arrhythmias

Common cardiovascular disease conditions, including diabetes, obesity, rheumatoid arthritis, aging, and congestive heart failure, have an association with immune dysregulation. Research investigating the immune response to cardiovascular disease and cellular injury is ongoing. Understanding how the immune system alters intracellular conduction, identifying treatment challenges, and highlighting potential areas of research may lead to better management of associated arrhythmias. Keefe et al.^1^ provided an in-depth review of how immune responses and inflammation contributes to cardiac arrhythmias. The authors portion out their review into two sections: the role of myeloid-derived cells in arrhythmias, followed by lymphoid-derived cells and their adaptive response to inflammation. In their review, the authors describe how specific immune cells influence atrial fibrillation (AF), postoperative atrial fibrillation (POAF), atrioventricular block (AVB), and ventricular tachycardia (VT).

### Atrial fibrillation

When investigating myeloid-driven cells, Keefe et al. explained that macrophages and monocytes compose most of the leukocytes within the heart and are imperative in combating tissue damage and infection. Monocytes themselves “play key roles in atrial fibrotic remodeling and predicted AF recurrence after catheter ablation,” they explain. Macrophages, derived from monocytes, have a complex relationship, through both cytokine secretion and direct electrical coupling, with cardiomyocytes via connexin 43 (Cx43). Pro-inflammatory macrophages can promote AF through cytokine release (like interleukin-1β), which can shorten the effective refractory period and contribute to re-entry circuit development. Anti-inflammatory macrophages are also involved, often driving pro-fibrotic remodeling, a key substrate for AF sustainability. There is a delicate balance between mitigating therapies and acknowledging that these same macrophages play an essential role in wound healing.

Shifting the focus to granulocytes, Keefe et al. identified several studies highlighting the neutrophil/lymphocyte ratio to be predictive of AF. Of the cytokines produced by neutrophils, myeloperoxidase may play an important role in atrial fibrosis. The authors also highlighted that there has been limited research on electrical coupling between neutrophils and cardiomyocytes.

The cellular complexity of arrhythmias continues when the focus shifts toward thrombocytes and erythrocytes. Ischemic injury drives the inflammatory process, which potentiates arrhythmias and thrombosis. High values of red blood cell distribution width, a marker of chronic inflammation, and polycythemia vera have been positively associated with AF. There is also a link between platelet activation and the incidence of AF through their release of pro-arrhythmogenic mediators such as serotonin and histamine. Additionally, several studies suggest that platelet-aggregation inhibitors reduce the incidence of arrhythmias.

It is relevant to mention that ischemic conditions increase cardiac mast cell content. These cells are responsible for histamine, leukotriene, and prostaglandin release during an immune response. The authors detail that mast cells have a dual involvement in both fibrotic and inflammatory processes.

### Postoperative atrial fibrillation

Although studies of human atrial tissue harvested during surgery have shown variable concentrations of macrophages, pericardial fluid collected within 72 h of surgery has demonstrated a higher concentration of macrophages in patients with POAF than in those without POAF. Further, monocyte-derived CCR2+ macrophages are the most prominently altered cell type in the mouse POAF model. Preoperative depletion of these CCR2+ macrophages in mice reduced the incidence of POAF. Shed mediastinal blood, rich in red blood cells and inflammatory mediators, has been implicated as a risk factor for POAF. Numerous reports indicate that this collection of focused inflammatory cells and factors at play contribute to POAF. Importantly, the authors remind us of the evidence from randomized controlled trials that POAF rates decrease with drainage of the pericardium into the pleural space. The presence of these immune and inflammatory mediators around the heart activates multiple cellular responses at the macrophage level and within the clotting cascade pathway.

### Atrioventricular block

AF is not the only arrhythmia impacted by inflammatory responses. Keefe et al. found increasing evidence that AV node conduction is dependent on a consistent level of macrophages, and depletion of these may indirectly result in decreased conduction and possible AVB. While limited in relationship to postoperative AVB, the authors report on a study that found low circulating neutrophil counts in pediatric cardiac surgical patients may be predictive of AVB.

### Ventricular arrhythmias

The complexity increases even more when discussing the role of macrophages in VT. Whether the presence of these cells predisposes to or protects from VT appears to be dependent on the subtype of macrophage and the level of activation. Limited studies also found conflicting evidence regarding cardiac neutrophil infiltration. Additional attention to their role in the acute phase of injury may be crucial in understanding their influence on VT. Thrombosis and the release of granule contents have been shown to promote arrhythmias, and there is a well-documented positive association between platelet activation and ventricular fibrillation.

## Mitochondrial dysfunction and fibrosis as cellular mechanisms of atrial fibrillation

While most therapies address the electrophysiologic symptoms and clinical sequelae of AF, its cellular basis still eludes us. The sophisticated research and cellular modeling reported by Son et al.^2^ offer a view into the cellular mechanisms that initiate and perpetuate AF. Using a unique three-dimensional (3D) model of human-induced pluripotent stem cell–derived cardiomyocytes, these biomedical researchers have examined mitochondrial dysfunction and associated atrial fibrosis at the cellular and genomic levels. Unlike monolayers of cardiomyocytes, this 3D cardiac organoid models human heart tissue in vitro and demonstrates features (electromechanical coupling, contractile responsiveness) that, under rapid pacing, enable investigation into mitochondrial dysfunction and cellular fibrosis at a molecular level.

Transcriptomic analysis comparing gene expression from human AF cardiac tissue to the in vitro organoid showed that gene-expression patterns related to cardiac fibrosis were strongly correlated between human AF tissue and the paced organoid model, validating its relevance for studying structural remodeling. In contrast, mitochondrial gene expression showed low correlation, suggesting heterogeneity in how mitochondrial regulation is modeled. Additionally, rapid pacing of the organoid induced significant functional remodeling, with a transition from synchronized rhythmic contractions to irregular chaotic beating. The organoids also experienced a nearly 50% reduction in maximum contraction amplitude and a >50% reduction in average contraction speed. These rapid pacing changes caused gene-expression changes indicative of pathological stress. Decreases in key maturity markers (*MYH7*, *CX43*) and upregulation of contractile component genes (*MYL7*, *MYL2*) may indicate compensatory or stress-related signaling.

Rapid pacing of the organoid results in a signaling mechanism that drives mitochondrial dysfunction and imposes severe metabolic stress on atrial cardiomyocytes (ACMs), quickly leading to mitochondrial failure and energy crisis. As metabolic overload increases, the production of reactive oxygen species (ROS) concordantly increases with direct damage to mitochondrial membranes and respiration impairment, further exacerbating the energy deficit. Sarcoplasmic reticulum calcium ATPase 2a, a key calcium regulator, experiences reduced expression/activity, leading to profound calcium deregulation with shortened action potential duration, a hallmark of AF electrical remodeling. Mitochondria, when stressed, release damage-associated molecular patterns (DAMPs). Both ROS and DAMPs trigger pro-inflammatory cytokines and initiate an inflammatory response that leads to electrical and structural remodeling. The high metabolic demand of fast pacing often depletes cellular ATP reserves and may accelerate cellular damage and subsequent apoptosis.

When these atrial organoids are co-cultured with fibroblasts, fast pacing simulates mechanical stretch and electrical overload that drive profibrotic signaling, thus creating a stable arrhythmogenic substrate. Fast pacing triggers the upregulation of transforming growth factor-β (TGF-β) in the cellular environment, the master regulator of fibrosis. TGF-β drives the differentiation of fibroblasts into highly active myofibroblasts, accelerating the synthesis and deposition of collagen I and III components in the extracellular matrix while initiating mRNA-mediated gene transcription of profibrotic factors, both resulting in structural remodeling (fibrosis).

## Mineralocorticoid receptor antagonism reduces postoperative atrial fibrillation

Danesh and fellow researchers^3^ at the Mayo Clinic described short-term (<30 days) and long-term (6 years) reductions in the occurrence of POAF with the use of preoperative mineralocorticoid receptor antagonists (MRAs) while exploring the association of mineralocorticoid receptor expression during atrial tissue stress. MRAs are known to reduce chronic AF.^4,5^ This study identified 19,042 non-AF cardiac surgery patients (age < 75 years), of whom 319 used MRAs preoperatively. This MRA group was propensity matched 1:3 (n = 298:894) based upon baseline and operative characteristics and comorbidities. To isolate the cardiac volume and electrolyte impacts of MRAs independent of their diuretic effect, a subgroup analysis matched MRA users against patients on diuretics other than MRAs. In parallel, left atrial tissue was collected from donor hearts without a history of AF immediately after procurement (n = 3) or after ex vivo perfusion with cold histidine–tryptophan–ketoglutarate solution with (n = 3) or without (n = 3) added canrenone (a water-soluble MRA). Left atrial tissue from AF patients (n = 3) was also collected and examined. Single-nucleus RNA sequencing was used to profile gene expression in the left atrial cells from these treated donors and from patients with chronic AF.

POAF occurred in 19.8% of MRA users at 30 days compared to 31.8% in non-MRA users (*P* < .001), with this difference persisting in the diuretic-only subgroup analysis. When examining the time to occurrence between the index procedure and 6 years, 9.2% of MRA users experienced a documented AF episode, while AF occurred in 18.9% of the non-MRA group (*P* < .001). Although the MRA users had a higher overall mortality rate, this mortality difference did not persist when the analysis was restricted to the cohort of propensity-matched diuretic users.

Atrial tissue analysis revealed that MRAs target stress pathways in cardiomyocytes and macrophages. Tissue from patients with chronic AF had an increased proportion of cardiomyocyte subtype 2 (CM2), which the authors describe as cells enriched for genes involved in inflammation, apoptosis, and mineralocorticoid receptor (MRc) signaling. In donor hearts preserved with canrenone, expression of MRc target genes in these CM2s was suppressed, whereas the same genes were elevated in the atria of patients with chronic AF. This suggests MRAs’ blunt activation of stress pathways in conduction-relevant atrial myocytes. Canrenone also downregulated a subset of genes generally upregulated by cold-preservation solution and that are normally upregulated in AF patients. Atrial macrophages treated with canrenone repressed the macrophage stress-response genes (eg, *FKBP5*, *ZBTB16*) that are otherwise elevated in chronic AF. The genes repressed by canrenone in macrophages matched genes upregulated in AF patients, indicating an anti-inflammatory effect.

## Our summary

The use of an in vitro cardiomyocyte organoid has enabled researchers to evaluate the stress of tachyarrhythmias using a clever reproducible model. The 3D organoid of Son et al. allows researchers to investigate markers of mitochondrial dysfunction and fibrosis at the molecular level. Study of the cardiomyocyte organoids confirms that oxidative stress leads to two intimately linked processes. Mitochondrial DAMPs and ROS signals activate profibrotic transcription factors in nearby fibroblasts. The resulting fibrosis further stiffens the tissue, increasing mechanical load, which, in turn, amplifies the stress on the mitochondria, perpetuating the cycle. Son et al. linked findings of mitochondrial dysfunction, mechanical remodeling, and structural fibrosis in this benchtop prototype to human AF atrial tissue. While organoid functional maturity and gene markers share consistencies with ACMs, this platform may not completely reflect AF in a human heart because it employs electrical stimulation. The researchers have, however, developed an investigational in vitro platform to test intracellular and genetic therapies, such as immune modulation and targeted genetic interventions.

Preoperative use of MRAs (most commonly spironolactone) is strongly associated with lower rates of POAF and late AF occurrences after cardiac surgery. The reduction in arrhythmias appears to be mediated by the suppression of MR-driven stress and inflammation in the atrium, especially within ACM subset 2. With gene-expression analysis, canrenone was found to reduce atrial stress responses by suppressing MRc target gene expression under conditions that replicate cardiac preservation and reperfusion. This suggests the future use of canrenone in cardiac preservation as a way of reducing POAF and indicates that cardiomyocytes are key targets for suppression of POAF through MRc antagonism with canrenone.

As we explore novel procedures and therapies designed for the management of cardiac dysrhythmias, comprehending cardiomyocyte physiology at the cellular and genetic levels remains crucially important. These reviews offer a brief glimpse into exciting areas of basic science research that may have future clinical and therapeutic relevance.

## References

[1] Keefe JA, Wang J, Song J, Ni L, Wehrens XHT (2025). Immune cells and arrhythmias. Cardiovasc Res.

[2] Son YH, Won J, Park YI, Park S-J, Jeong GJ (2025). Mitochondrial dysfunction and fibrosis in atrial fibrillation: molecular signaling in fast-pacing organoid models. J Ind Eng Chem.

[3] Danesh S, Khan F, Chopko T (2025). Mineralocorticoid receptor antagonism reduces atrial arrhythmias post-cardiac surgery and attenuates atrial stress responses to cardioplegic arrest. J Am Heart Assoc.

[4] Liu T, Korantzopoulos P, Shoa Q, Zhang Z, Letsas KP, Li G (2016). Mineralocorticoid receptor antagonists and atrial fibrillation: a meta-analysis. Europace.

[5] Neefs J, van den Berg NW, Limpens J (2017). Aldosterone pathway blockade to prevent atrial fibrillation: a systematic review and meta-analysis. Int J Cardiol.

